# Toward Rational Design of Imprinted Proteins Based on Albumins: Computational and Experimental Studies

**DOI:** 10.3390/polym18111280

**Published:** 2026-05-23

**Authors:** Polina M. Ilicheva, Alexander L. Kwiatkowski, Ivan A. Reshetnik, Kirill Y. Presnyakov, Ilya E. Menyailo, Mikhail V. Pozharov, Pavel S. Pidenko, Yulia B. Monakhova, Olga E. Philippova, Natalia A. Burmistrova

**Affiliations:** 1 Institute of Chemistry, Saratov State University, Saratov 410012, Russia; reshetnikia@sgu.ru (I.A.R.); menyilo.ilya1997@gmail.com (I.E.M.); pozharovmv@gmail.com (M.V.P.); pidenkops@sgu.ru (P.S.P.); naburmistrova@mail.ru (N.A.B.); 2 Physics Department, Lomonosov Moscow State University, Moscow 119991, Russia; kvyatkovskij@physics.msu.ru (A.L.K.); phil@polly.phys.msu.ru (O.E.P.); 3 Department of Chemistry and Biotechnology, University of Applied Sciences Aachen, D-52428 Jülich, Germany

**Keywords:** molecular imprinting, imprinted proteins, bovine serum albumin, 4-hydroxycoumarin, isothermal titration calorimetry, NMR–DOSY, molecular dynamics, metadynamics, molecular docking

## Abstract

Imprinted proteins (IPs) are promising materials for producing artificial alternatives to natural recognition systems (antibodies, aptamers, etc.) due to their high sorption properties and specificity. However, contemporary understanding of the imprinting process at the atomic level is rather limited, which hinders the rational design of more efficient IPs. In this paper, we use computational modeling to provide a description of fundamental principles of protein imprinting at the atomic level. We have modeled several potential associates between the protein matrix and template molecules that form during the imprinting process up to the addition of the cross-linking agent. We used bovine serum albumin (BSA) as the protein matrix and 4-hydroxycoumarin (4–HC) as a molecular template. In combination with computational modeling, extensive experimental analyses including isothermal titration calorimetry (ITC) and NMR spectroscopic methods (^1^H NMR and diffusion-ordered NMR spectroscopy (DOSY)) were used to evaluate the potential efficiency of imprinted BSA. This study represents a step toward the future rational in silico design of IPs.

## 1. Introduction

Molecular imprinting is a method that found widespread application in different areas of modern science and technology [1,2]. One of the key factors in the development of this technique is the need to create a synthetic alternative to natural molecular recognition systems, such as enzymes and antibodies [3]. The use of organic solvents and the difficulty of obtaining identical polymer structures can cause several problems related to the environmental risks of imprinting and the reproducibility of the physicochemical properties of obtained imprinted polymers. Therefore, protein molecules serve as a potential alternative to artificial polymer matrices for imprinting. Artificial biosynthetic receptors are called imprinted proteins (IPs or protein-based nano-molecularly imprinted polymers) and are most similar in nature to antibodies [4]. The principle of producing such receptors (also known as conformational modification or bioimprinting) is based on fixation of altered conformational structure of the protein matrix after its association with template molecules. There are two main approaches to “freezing” the structure of a protein matrix in imprinting: transfer to an organic solvent and intramolecular chemical fixation using a cross-linker.

Use of aqueous solvents and the possibility of including IPs in immunochemical assays are key advantages of IPs, and they can be achieved by intramolecular chemical fixation. Such IPs are successfully used for biocatalysis [5] and as an element of molecular recognition in the development of test systems [6]. Analytical characteristics of test systems with such receptors are comparable to those of commercial immunochemical systems. Although there are examples of IP-based test systems [6,7], they have one significant drawback—insufficient understanding and level of research into the nature of the imprinting process and, consequently, a lack of generic and effective approaches for obtaining IPs with specified properties. The key stages of IP production have been postulated [5], but their necessity and the extent to which they influence the properties of the final product are not fully understood. A fundamental explanation of these stages requires a detailed description of the process of IP formation at the molecular level. In this regard, computational techniques are a powerful tool for studying the mechanisms of the imprinting process and providing enormous opportunities for the rational design of IPs, including the prediction of key properties relevant to adsorption processes and selectivity characterization.

Various computational approaches, such as quantum mechanics (QM), molecular mechanics (MM), or molecular dynamics (MD), have been actively used to simulate ligand–protein systems over the last 5 years for molecularly imprinted polymer design [8] and currently form the basis for IP design. There are several interesting studies dedicated to estimating the binding affinity of various monomers with proteins [9,10,11,12]. The uniqueness of these studies stemmed from the analysis of proteins as a template for imprinting and the application of molecular docking as a theoretical method. However, inadequate estimations of molecular flexibility and solvent-mediated binding, which play a crucial role in thermodynamics and kinetics of molecular recognition, are significantly limiting reliability with respect to the predicting ability of this method [13]. Though most software packages can take ligand flexibility into account, protein flexibility still remains a challenging issue [14]. Fluctuations of the protein can be considered before, during, and after the docking procedure using protein conformation data from various crystallographic and NMR databases or by calculating them via computational methods, but these representations are divorced from the binding process itself. This can alter the final structure of the complex and its predicted binding affinity, limiting the predictive power of these methods.

In practical terms, searching for a protein matrix that has the best adsorption properties and selectivity for templates is the main task for obtaining the most efficient IPs. To find the appropriate optimal conditions, we must carefully study the fundamental interactions between the protein matrix and the template, since the underlying binding mechanism remains unclear in each individual case. Generally, the focus of IP development is related to simulating protein–template interactions through molecular docking, but thorough examinations of the mechanism of association require studying the behavior of both proteins and templates under chosen synthesis conditions. Since any protein molecule can react strongly to changes in external conditions, one of the key aspects of molecular modeling used for IP development is related to the analysis of conformational dynamics of the protein matrix during the imprinting process. The approximation of possible side processes occurring in the template before its association with a protein can be performed via MM or QM approaches. A variety of existing molecular modeling methods allows us to describe some processes, but each system requires its own set of methods most suitable for simulating its specific features.

In a previous study [15], we analyzed several IP systems and were able to provide practical recommendations for optimizing synthesis conditions through a computational approach due to the rethinking of the concept of IP preparation in line with automated protocols used in synthesis. The most effective system, in this case, is determined in accordance with the number and nature of template-specific binding sites. Thus, computer simulations of various templates reduce the labor costs of building an IP library. However, confirmation of the proposed computational principles is impossible without coordination with experimental studies, including biophysical methods. The ambiguity of IP synthesis and the limitations of experimental methods significantly complicate our understanding of the nature of the process. Therefore, it is necessary to use experimental methods to study the key stages of IP production to increase our understanding of the processes and interactions involved and to potentially validate modeling results.

In this study, we focused on the mechanism of protein imprinting, using a combination of experimental and computational techniques to provide a more exhaustive characterization of structural changes in the protein matrix and protein–template binding interactions. We expanded and improved the previously proposed IP design strategy [15] and, for the first time, compared the calculated structural parameters of BSA before and after the addition of template molecules (4-hydroxycoumarin, 4–HC) with experimental results. We used a combination of computational methods, such as molecular docking, molecular dynamics, and well-tempered metadynamics, to explain the process of association between protein matrix and template molecules at the atomic level. To study the formation of complementary binding sites in the protein structure at different pH values, we used several experimental techniques: isothermal titration calorimetry, NMR, and dynamic light scattering (DLS). The present work primarily focuses on the mechanistic investigation of BSA–4–HC interactions during the imprinting process, while the performance data for the final IPs, such as binding efficiency, sorption capacity, and selectivity, and comparisons with similar parameters of non-IPs were provided in a previous study [16].

## 2. Materials and Methods

### 2.1. Materials

BSA and 4–HC (98%) were purchased from Merck (Merck KGaA, Darmstadt, Germany). All solutions were made using double-distilled water treated via a Milli–Q system (Millipore, Burlington, MA, USA). The purity of all other chemicals was analytical grade. The pH was adjusted with HCl (0.1 M) and NaOH (0.1 M) solutions.

### 2.2. Samples Preparation and NMR–DOSY Measurements

For NMR measurements, approximately 40 mg of BSA was dissolved in 1 mL of H_2_O/D_2_O (+ 0.1% TSP as the internal standard) taken at a 9:1 ratio. The pH was adjusted using the HCl or NaOH solution to the following values: 2.2; 3.3; 4.2; 4.7; 5.8; 7.7; and 9.0. NMR spectra were measured within no more than 30 min after sample preparation to avoid degradation of solutions. To evaluate the robustness of the method, initial weight of BSA was varied between 10 mg and 100 mg (7 points). To evaluate the precision of measurements, several samples were prepared and measured in triplicate.

To experimentally investigate the imprinting process, 40 mg mL^−1^ of BSA stock solution was prepared in H_2_O/D_2_O taken at a 9:1 ratio. The pH was adjusted to 3.0 with the HCl solution, and the mixture was shaken for 10 min. Then, 60 μL, 30 μL, and 3 μL of 4–HC stock solution (1.3 mg mL^−1^ 4–HC in methanol-d4) was added to 3 mL of this BSA stock solution to produce Mixtures **1A**–**1C**. These three mixtures were shaken for 20 min, and an aliquot of 0.6 mL was taken from each mixture. Afterwards, the pH of each solution was adjusted to 8.0 using a NaOH solution, and an aliquot of 1 mL of each solution was taken to produce Mixtures **2A**–**2C**. Then, 20 μL, 10 μL, and 1 μL of glutaraldehyde (0.5%) were added to 1 mL of Mixtures **2A**–**2C** to produce Mixtures **3A**–**3C**. After this, NMR spectra of the 0.6 mL Mixtures **3A**–**3C** were measured immediately. Mixtures **A**–**C** had varying concentrations of 4–HC in order to correct the analytical signal. All measurements were carried out at room temperature.

High-field ^1^H NMR measurements were performed using a Bruker Avance III 500 MHz spectrometer (Bruker Biospin, Rheinstetten, Germany) with a BBO cryo probe equipped with a Bruker Automatic Sample Changer for 60 samples at 297 K. ^1^H NMR spectra were recorded with a 1D NOESY pulse program with water presaturation and spoiler gradients applied during relaxation using 32 scans. In total, 132k points of data were acquired with a pulse width of 11.64 s, a receiver gain of 69.5, an acquisition time of 5.4526 s, and relaxation delay of 5.0 s. Then, 2D diffusion-ordered NMR spectroscopy (DOSY) was performed using a DOSY pulse sequence with water presaturation through longitudinal eddy current delay with bipolar gradient pulse pair and 2 spoil gradients. The length of the gradient pulse (δ) was set to 1400 μs, and diffusion time (Δ) was set to 0.05 s. Four scans provided enough sensitivity (this parameter was varied between 2 and 16). The measurement took only 15 min for one sample. The data were recorded automatically under the control of ICON–NMR (Bruker Biospin, Rheinstetten, Germany).

NMR spectra were processed manually in MestReNova version 14.1.2 (Mestrelab Research, Santiago de Compostela, Spain). Spectra were exponentially apodized with a factor of 1.0 Hz. For comparability, all spectra were referenced to TSP at δ 0.0 ppm. The phase and the baseline were corrected manually for the entire spectrum. The Dynamic Center (Bruker Biospin, Rheinstetten, Germany) was used for the processing of DOSY spectra. The 2D plots show diffusion coefficient values D in [m^2^ s^−1^].

### 2.3. Isothermal Titration Calorimetry

Isothermal titration calorimetry (ITC) was used to obtain the association thermodynamic parameters ($\Delta H_{\mathrm{as}}$ and $\Delta S_{\mathrm{as}}$), binding constant K, and the number of binding sites (*n*) for the 4–HC imprinting into the BSA at different pH values. The measurements were carried out using a NanoITC isothermal titration calorimeter (TA Instruments, New Castle, DE, USA) at 298 K. In total, 50 μL of the 4–HC solution in the syringe was titrated into 170 μL of the BSA solution in the sample cell at a mixing rate of 300 rpm. In total, 25 injections of 2 μL were made. The interval between injections required for mixture equilibration was 300 s. All solutions were degassed at 380 Torr and 298 K for 40 min before the experiments. The raw ITC data of 4–HC titration into BSA were corrected by subtracting the thermograms of 4–HC titration into the corresponding buffer solution at the same pH. The corrected data were used to create a titration plot showing the observed enthalpy change as a function of the 4–HC:BSA molar ratio. The first peak of the thermograms was ignored. The titration plots were fitted with the multiple-site titration model in NanoAnalyze 3.12.0 software (TA Instruments, New Castle, DE, USA) to estimate values of ΔH, ΔS, *K* and *n*.

### 2.4. Dynamic Light Scattering

The hydrodynamic diameter measurement of proteins at different parameters was carried out using ZetaSizer Ultra Red Label (Malvern Panalytical Ltd., Malvern, UK). Three (n=3) measurements were performed at 298 K in 10 mg mL^−1^ of aqueous solution. Knowing the actual mean hydrodynamic diameter of the particles (i.e., globules) at certain pH values and protein concentrations, it is possible to calculate the diffusion constant (*D*) by means of the Stokes–Einstein equation (Equation (1)), where $k_{B}$ is the Boltzmann’s constant, *T* is the thermodynamic temperature, η is the viscosity of the medium, and $r_{h}$ is the particle or globule radius.

(1) $$D=\frac{k_{B}T}{6\pi \eta r_{h}},$$

### 2.5. Computational Details

#### 2.5.1. Ligand and Protein Preparation

The crystallographic structure of BSA (PDB ID: 4F5S) at a resolution of 2.47 Å was downloaded from the Protein Data Bank database [17]. For calculations, we considered the monomeric form of BSA. Protein structure preparation included the addition of hydrogen atoms and missing amino acid side chains, removal of solvent molecules, and adjustment of the protonation state. Protonation and histidine tautomer states were adjusted according to the acidity constants of the amino acid residues to simulate specific pH levels using the PROPKA algorithm [18]. The geometric parameters of the protein and ligands were calculated with an amber99sb-ildn force field [19]. All ligands were parameterized with acpype [20]; atom point charges for all ligands were derived from B3LYP/6–31G* ab initio calculations using psiresp [21].

#### 2.5.2. Molecular Docking

Binding site prediction was performed using SiteMap (Schrödinger, San Diego, CA, USA) [22,23] and AutoDock Vina (v 1.2.3) [24]. Ligand docking was performed using Glide implemented in the Schrödinger Suite 2021-2 program [25,26,27,28] with the following conditions: inflexible protein and flexible ligand, 15 Å grid matrix size. Docking solutions were ranked by evaluating the following calculation parameters: docking score (based on GlideScore with penalty exclusion) and the model’s energy value parameter (Emodel), including the GlideScore value, energy of unbound interactions, and energy parameters spent on the formation of compound stacking at the binding site. Further, ligand–protein complexes were placed into the orthorhombic system, with a 13 Å buffer zone starting from the surface of the protein. Systems were filled with TIP3P water in NVT and NPT environments. The recorded dynamics simulation periods were 300 ns (at 298 K). We ran three replicate simulations for each system. The protocol for simulation system’s preparation included preliminary minimization and equilibration stages. As the solvent was simulated explicitly in MD, binding affinity was estimated through conventional Molecular Mechanics Generalized-Born Surface Area (MM–GBSA) technique [29].

#### 2.5.3. Molecular Dynamics and Metadynamics Simulations

MD simulations were performed with GROMACS 2024.3 [30,31,32] and PLUMED 2.9.3 [33] software packages using the hardware of the shared research facilities of HPC computing resources at the Saratov State University (Saratov, Russia). The system was placed in a cubic box with periodic boundary conditions and solvated with TIP3P water molecules. Na^+^ and Cl^−^ ions were added to neutralize the net charge. Systems were minimized with 5000 steps of steepest descent. System equilibration consisted of several steps. NVT and NPT run with heavy atoms positionally restrained at 1000 kJ mol^−1^ nm^−2^ was performed for 1 ns. Velocity rescale thermostat was utilized for temperature coupling [34]. The reference pressure was 1 bar, and it was simulated by a stochastic barostat [35]. A time step of 2 fs was used in all steps.

To overcome the energy barriers and obtain stable conformation, BSA molecules were sampled used well-tempered metadynamics. Two collective variables were used: the distance between centers of mass of the D1 and D3 domains and an angle between these centers. Metadynamics potentials of 1 kJ mol^−1^ with a width of 0.04 nm and 0.02 rad were applied every 500 steps. The bias factor was set to 20. This simulation used 6 walkers, each accumulating 1500 ns of trajectory, thus accumulating a total of 9 μs of trajectory.

## 3. Results

Imprinting of proteins is based on reversible changes in the three-dimensional structure of a protein induced by non-covalent interactions with a ligand in mild denaturing conditions [5]. After these changes, a protein shows altered binding properties. The standard technique of imprinting described by Liu et al. [5] consists of a few basic stages: (i) the protonation of amino acid residues of a protein under acidic conditions; (ii) addition of template molecules to form new binding sites; (iii) stabilization of the new conformation via cross-linking of the protein; and (iv) removal of template molecules from the protein matrix. First, we evaluate the physical and chemical parameters of free BSA and BSA–4–HC associates under synthesis conditions via experimental techniques. The second part is devoted to molecular simulation in time intervals, enabling a description of protein–template interactions during the imprinting process. Attention is paid to the conformational states of BSA and assessments of the stoichiometry of the interaction between 4–HC and BSA.

### 3.1. Dynamic Light Scattering

A change in the pH of the medium results in ionization of amino acid residues, thus disrupting the electrostatic interactions that stabilize the protein structure. This leads to conformational changes (unfolding) in the molecule, exposing internal hydrophobic regions and providing additional potential binding areas. At the same time, exposure of the hydrophobic surface may mediate the dimerization of BSA because the protein tends to bury exposed amino acids by binding to another monomer. Therefore, we first assessed potential aggregate formation in BSA solutions with varying pH levels.

The pH changes induce protonation and deprotonation of the dissociable groups of proteins—the carboxylic (aspartic acid and glutamic acid) and amine (lysine, arginine, and histidine) side groups. Due to the charge density changes, the electrokinetic properties of the proteins also vary with pH. We have measured the changes in the electrokinetic potential (ζ-potential) of albumin (Table 1). The found protein charges are highly positive in acidic media and negative in alkaline media. We have also studied how the average hydrodynamic globule size changes as a result of pH variations (Table 1). The approximate hydrodynamic radius ($R_{h}$) of native BSA is 3.88 nm [36]. The average $R_{h}$ does not change significantly with the protein concentration from pH 4.5 to pH 8 but increased at pH 3. The observed results are in good agreement with the previously reported $R_{h}$ of BSA at various pH conditions obtained by DLS [36,37]. The net charge of the BSA globules around the isoelectric point (${\mathrm{pH}}_{i.e.p.}$) is close to zero; thus, we can observe the formation of a more contracted, rigid phase. This explains why the volume fraction of the protein in the solution is rather low even in the higher concentration range. At pH 3, the globules form an expanded phase, and the more easily accessible charged groups are highly solvated (i.e., hydrated). As a result, the volume fraction of the protein in the solution increases, leading to a decrease in the diffusion constant upon an increase in the protein concentration. As shown in Figure S1, small-scale aggregation (∼100 nm) did occur for the partially unfolded form of BSA. Thus, the increase in intensity at size values of ∼100 nm indicates that partially unfolded BSA monomers at pH 3.0 should coexist in solutions with higher-order aggregates. Scanavachi et al. [38] suggested the coexistence of monomers (∼70–80%) and dimers (∼15–25%) at similar pH values based on the results of high-performance liquid chromatography assays. Thus, at a given concentration of 10 mg mL^−1^, we assumed that partially unfolded monomers are the dominant form of BSA in the solution. Assessing the specific influence of GA cross-linking showed that the hydrodynamic diameter of BSA increases slightly, while the ζ-potential remains at the same level (Table 1). Maintaining colloidal stability is achieved through the hydration shell, electrostatic repulsion, and steric factors. The imprinting process requires the use of cross-linking agents and pH changes, which can disrupt the balance of forces. The disruption of colloidal equilibrium inevitably leads to coagulation (aggregation) of particles and loss of functionality. However, we showed that BSA predominantly retains its size even after cross-linking.

### 3.2. Diffusion-Ordered Spectroscopy Studies

**Figure 1:**
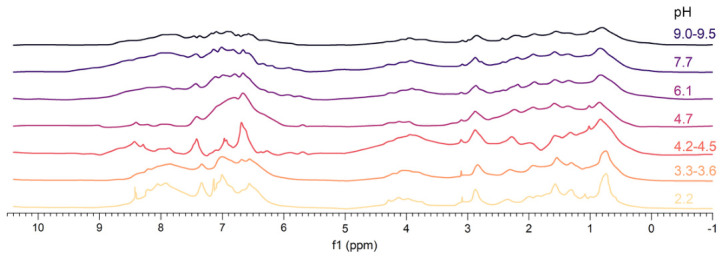
DOSY NMR spectra of free BSA at different pH values.

The DOSY NMR spectra of proteins record proton signals corresponding to mixture components separated by their diffusion coefficients. Typically, signals from amide protons, side-chain protons, or alpha protons that decay with increasing magnetic field gradients are used to determine the protein’s size and hydration radius [39]. The DOSY NMR spectra of free BSA at different pH values are provided in Figure 1 and Figure S2. The results of the DOSY NMR measurements of BSA are reported in Table 2. For BSA molecules in water solutions, it can be concluded that the size of molecules decreases when pH rises from 2.2 to 3.3. Further size reductions occur at pH 4.2. Afterwards, it remains constant within the pH interval of 4.2–7.7. At pH 9.0–9.5, the BSA molecule has the biggest volume. The results confirm that the BSA size grows when the solution’s pH varies from ${\mathrm{pH}}_{i.e.p.}$. The hydrodynamic mean diameter of globules increases with increasing alkalinity or acidity of the solution, while the relative diffusion coefficients decrease.

NMR measurements of diffusion coefficients using DOSY NMR spectroscopy (Figure S3, Table S1) showed that, after the addition of 4–HC to BSA, the size of the BSA molecule remains the same as it was at pH 3 (Mixtures **1A**–**1C**). However, after adjustments at pH 9, the molecule size decreases (Mixtures **2A**–**2C**). The addition of GA to these mixtures leads to a further decrease in BSA molecule size (Mixtures **3A**–**3B**) or it stays constant at small GA concentrations (Mixture **3C**). This fact confirms that IPs have monomer forms without protein aggregates and intermolecular cross-linking.

Additionally, these samples were studied via IR-Fourier spectroscopy. Detailed descriptions of the obtained results are shown in the Supplementary Materials (IR Spectroscopy Section, Figure S4).

### 3.3. Isothermal Titration Calorimetry

**Figure 2:**
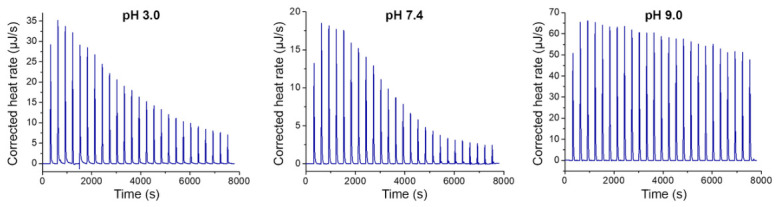
The ITC raw data for titration of the 0.5 mg mL^−1^ solution of 4–HC into the 5 mg mL^−1^ aqueous solution of BSA and buffer solutions at various pH levels at 298 K.

**Figure 3:**
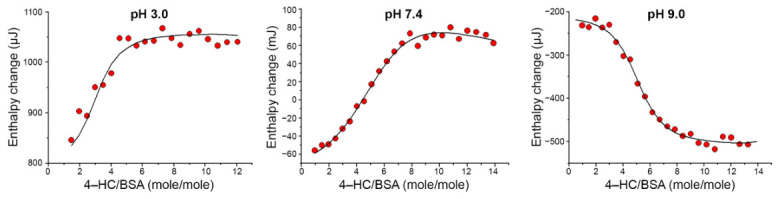
The binding isotherms for the titration of a 0.5 mg mL^−1^ 4–HC solution into a 5 mg mL^−1^ aqueous solution of BSA at various pH levels at 298 K, obtained after the correction of ITC raw data with thermograms of the 4–HC dilution in the corresponding buffer solutions (Figure S5).

Figure 2 demonstrates the ITC raw data for the titration of 4–HC in aqueous solutions of BSA and corresponding buffer solutions at different pH values. The titration plots obtained after subtracting the thermograms of the 4–HC dilution with buffer solutions at required pH values (3.0, 7.4 or 9.0) are presented in Figure S5, while the binding isotherms are provided in Figure 3. The binding isotherms cannot be fitted using a simple one-site binding thermodynamic model that describes the interaction of a ligand with a macromolecule with binding sites of the same type [40]. For this reason, more complicated multiple-binding-site models that account for the interaction of ligands with several types of binding sites [41] have been applied (Figure 3). The plots can be well fitted using a multiple-binding-site model (Figure 3). The fitting parameters are summarized in Table 3. At all studied pH values, the titration curves show that there are two processes with different association constants: $K_{1}$ and $K_{2}$ (Table 3). Both processes are spontaneous because they are characterized by negative values of Gibbs free energy ΔG. In all cases, $K_{1}$ is two orders of magnitude larger than $K_{2}$, indicating a much higher probability of the first process.

Let us first consider the first and most dominant process. At acidic pH levels, its ΔH and ΔS values are positive (ΔH>0 and ΔS>0), which suggests that the process is entropically driven as is typical for hydrophobic interactions [42]. Therefore, under these conditions, hydrophobic interactions are primarily involved in the imprinting of 4–HC into BSA [42]. This is consistent with the hydrophobic nature of 4–HC, which does not dissolve in water under acidic conditions [43]. At pH 3, the hydrophobic interactions between 4–HC and BSA lead to complex formation with a stoichiometry of n≈1–2 (Table 3).

At pH 7.4, ΔH becomes negative (ΔH<0 and ΔS>0). So, the first process becomes driven both by the gain of enthalpy and entropy [44]. According to S. Siddiqui et al. [42], in this case, the binding of 4–HC to BSA is governed by electrostatic interactions, i.e., the attraction between the deprotonated hydroxyl group of 4–HC [45] and positively charged areas of the BSA surface. This is consistent with the p$K_{a}$ value of 4–HC (4.1 [45]) and the isoelectric point of BSA at pH 4.5 [46]. At pH 9, the thermodynamic characteristics of the first process remain the same as at neutral pH (Table 3), ΔH<0 and ΔS>0, suggesting that the driving force of the 4–HC binding to BSA is still the same—the electrostatic interactions between the charged groups of 4–HC and BSA. The higher affinity of 4–HC and BSA at pH>7 is also evidenced by the rise in the stoichiometric coefficient of the complex to n≈5, indicating that more sites of BSA become available for the interaction with 4–HC.

Now let us consider the second process. At pH 3 and 7.4, it is entropically driven, as the ΔH and ΔS values are positive (ΔH>0 and ΔS>0), which is indicative of hydrophobic interactions. We believe that the second process represents the hydrophobic aggregation of excess 4–HC molecules that are not bound to BSA due to their low solubility in water both at acidic and neutral pH [43]. The second process of aggregation was also observed with respect to ITC by M. De and co-workers [47] during the complex formation of BSA with oppositely charged modified gold nanoparticles at pH = 7.4. This process was also shown to have a 6–7 times higher stoichiometric ratio *n* than the first process of binding of nanoparticles to BSA [47]. At pH 9, both ΔH and ΔS change their signs, ΔH<0 and ΔS<0, indicating the change in the nature of the second process. Negative values of both ΔH and ΔS can be attributed to the presence of hydrogen bonding [42]. One can speculate that these changes in thermodynamic parameters together with the increase in the stoichiometric coefficient *n* (Table 3) could be the result of multiple interactions between the excess charged 4–HC and water molecules during 4–HC dissolution in water at high alkaline pH. We have also considered the possibility of the hydrolysis of template molecules in an alkaline medium that could lead to the opening of the lactam ring, and we have found that the 4–HC molecule, unlike coumarin, does not undergo hydrolysis (exp. data are detailed in the Supplementary Materials, Figure S6).

### 3.4. Molecular Modeling

**Figure 4:**
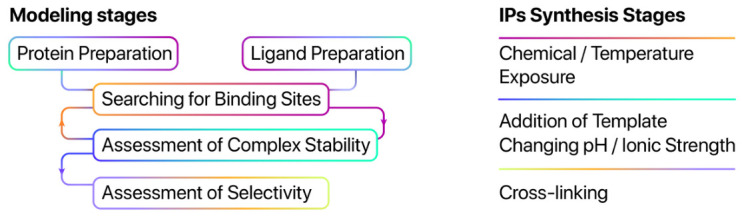
Schematic representation of simulation protocol.

Recently, we have published the results of a study of interactions between relatively small organic molecules (e.g., amino acids and their derivatives, as well as various toxins) interacting with BSA, which may be promising for understanding the mechanisms of the bio-molecular association of IPs [15,48]. There, we proposed a new protocol that combines standard docking calculations with free-energy estimations obtained from molecular dynamics and metadynamics (Figure 4). This may serve as a step forward to the development of a tool capable of optimizing synthesis conditions and IP development.

We applied this protocol to the study of 4–HC binding to the BSA. Metadynamics simulations were initially used to calculate the protein structure in aqueous solutions at pH 3.0. Standard docking calculations were then used to provide a first guess of the putative binding regions of template molecules on the protein’s surface. Subsequent MD simulations were carried out to estimate the stability of the ligand–protein complex. Since the state of the protein and ligand could change during synthesis, the cycle of searching for binding sites and assessing the stability of the complex was repeated for each variation in synthesis conditions during the imprinting process before the addition of a cross-linking agent.

BSA is the most common and extensively utilized protein in both computational and experimental studies. BSA is composed of 585 amino acid residues generally grouped into three homologous domains (1, 2, and 3) resulting in a molecular mass of 66.4 kDa [49,50]. This protein is relatively stable due to its 17 disulfide bridges, and its isoelectric point is around ${\mathrm{pH}}_{i.e.p.}$ 4.5–5.5 [51]. The starting point for understanding the mechanism of the formation of IPs is a description of the protein structure at the first stage of synthesis, namely, protonation in an acidic environment. Though each protein has an optimal pH range where its binding properties are maximized, we focused on the conventional synthesis conditions with pH 3 at the first stage. BSA immersed in aqueous solutions undergoes reversible conformational rearrangement as a result of changes in the solution’s pH, leading to the formation of different conformers—E-form (extended) at pH<3, F-form (fast) at pH 3–5, N-form (native) at pH 5–7, B-form (basic) at pH 7–8.5, and finally, A-form (aged) at pH>8.5 [37]. In this regard, we investigated various protein structures corresponding with changes in the solution’s pH to better understand the structural properties of BSA during IP synthesis.

### 3.5. Conformational Study of Free BSA

Recently, it was shown that MD simulations provide detailed information on the structure of BSA in acidic environment, where electrostatically induced partial denaturation was observed, associated with the displacement of molecular domains relative to each other [15,38,52,53,54]. Although the models were well supported experimentally, computational time on the scale of tens of nanoseconds is insufficient for sampling large-scale conformational changes that typically occur within hundreds of microseconds. In most cases, the intermediate conformational states are unstable but give insights into the protein’s structure [53]. However, the analysis and characterization of conformational structures of these protein intermediates are very challenging and difficult. In this regard, we performed a metadynamics study to reconstruct the energy profile of two presumably linked processes at pH 3—protein protonation and domain displacement. For these, the complete BSA structure in monomeric form was considered.

**Figure 5:**
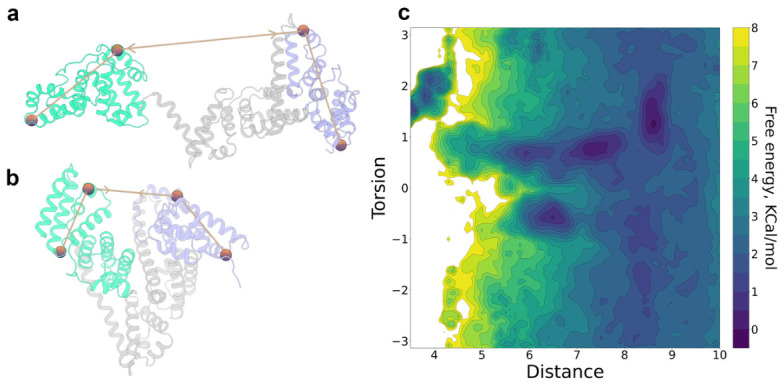
BSA’s simulated structure showing the state at the major energy minimum at pH 3 (**a**); native structure of BSA (**b**); free-energy map of simulated BSA structure at pH 3 for D1–D3 domain distances and the torsion angle of twist coordinates (**c**). Interdomain distance was calculated as a line connecting the centers of mass of the D1 and D3 domains.

Using simulation, free-energy surfaces were evaluated for free BSA at pH 3. The partitioning of free energy into multiple minima (Figure 5) demonstrates the existence of several potential metastable states. We observed three energy minima (ΔG) with low transition energy barriers (ΔΔG) (Figure 5). Transitions from the native state to a partially unfolded state are characterized by a ΔΔG of 5.85 kcal mol^−1^. We compared the geometric parameters of the conformational state of the major minimum with parameters of the F-form of BSA and found that the BSA structure took a certain partially unfolded form, where complete linearization of the molecule was not observed. Twist of domains increases along with the interdomain distance, and the molecule acquires the possibility of formation of additional hydrophobic binding sites (Figure 5). The interdomain separation resulted in an increase in the solvent’s accessible surface area from 285 nm^2^ at pH 7 to 336 nm^2^ at pH 3. It should be noted that not all proteins change their conformation in similar conditions and that they may not significantly change their hydrophobic surface area compared to BSA. In this regard, the use of an acidic environment is not necessary for any proteins in IP synthesis and may be redundant. At the same time, the protein’s surface area can be increased by temperature changes, and this approach can be considered an alternative for pH changes.

#### Conformational Study of BSA–4–HC Associate

The second key stage of imprinting is the association of template molecules with proteins. Obtaining imprinted materials using a dummy template is a well-known “green” imprinting approach [55,56]. In this case, a dummy structural analog or synthesized derivative is used as a replacement for the toxic target template’s molecules. In this study, we analyzed the previously prepared sorbent [16] that used 4–HC as a dummy template to obtain imprinted BSAs specific for zearalenone as a model system. 4–HC was chosen due to the absence of side processes that could complicate the description of the BSA–template interaction in this system and the absence of 4–HC ionization under imprinting conditions [57], allowing us to evaluate the effect of environmental acidity on the structure of the associate with BSA. Since IPs have group specificity, the use of 4-HC as a template suggests group specificity for coumarins. Due to their unique optical, biological, and photochemical properties, natural and synthetic coumarin derivatives find a wide range of applications [58]. Coumarins can be used as fluorescent markers for imaging, in addition to being used as targeting agents and active components in drug delivery systems. The design and incorporation of coumarin-based sensors enable sensitive and selective detection, facilitating advancements in environmental monitoring, medical diagnostics, and chemical analysis [59].

Based on the obtained conformational state of BSA, ligand–protein interactions were studied in the second stage of synthesis. A blind docking protocol was used to identify binding sites on the full surface of the protein matrix after the addition of template molecules at pH 3.0. Additionally, we verified the formation of binding sites using the Site Map plugin and Boltz-2 neural network model [60]. Analyses of the modeling results showed the presence of six potential binding sites on the BSA’s surface (Figure S7a). According to docking results, the energy values characterizing ligand affinity are approximately the same (Table S2). MD simulations were then used to characterize the retention efficiency of ligands at all binding sites, describing the state of the system for 300 ns. We suggest that 4–HC binding occurs in two of the likely binding sites (Figure S7b). The stable retention period in two potential binding sites is observed during the whole period of simulation (Figure S7c); both binding sites are saturated with hydrophobic amino acids (Table S2). This observation is consistent with ITC results. Four other ligands are retained at their binding sites for only 150 ns out of 300 ns, and they move into the solution phase after that.

**Figure 6:**
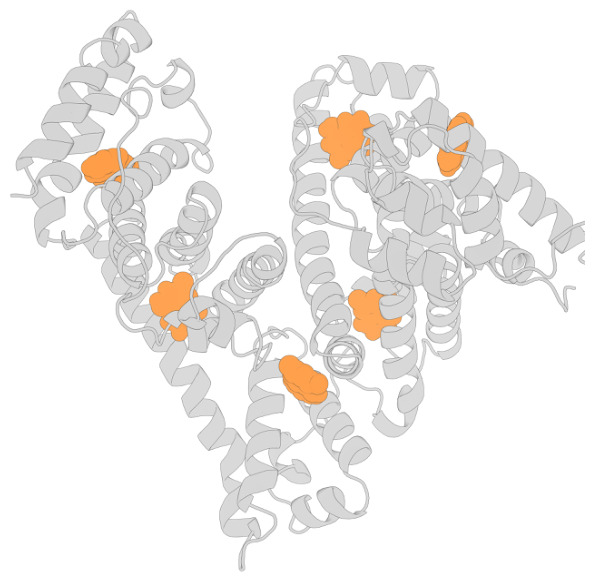
The final structure of BSA–4–HC associates during the synthesis process before fixing the protein matrix.

The next step in the imprinting process is a transition to pH 9 to fix the new state of the protein matrix, and this may be accompanied by the destruction of ligand–protein interactions in existing binding sites and the formation of new interactions at different binding sites. Further MD simulation confirmed the stability of two binding sites formed at pH 3, when the solution’s pH was changed to 9 (Figure S8). Blind docking was performed to evaluate the formation of additional binding sites at pH 9. Analyses of the modeling results showed the presence of six new potential binding sites on the BSA’s surface (Figure S8a). We observed the formation of powerful π-cationic interactions (Table S2), thus confirming our conclusions derived from the ITC assay; namely, that electrostatic interactions are the driving force behind the binding of 4–HC to BSA. Analyses of the MD trajectory of the complex showed that the retention time remained throughout the simulation period for all ligands (Figure S9b). As a result, there were a total of six binding sites during the imprinting process (Figure 6 and Figure S9a).

According to the results obtained via ITC, the number of binding sites (*n*) was 1–2 and five at pH 3 and 9, respectively. According to the modeling results, the number of binding sites (*n*) was two and six at pH 3 and 9, respectively. The appearance of additional ligand binding regions at pH 9 can be explained by the conformational rearrangement of the protein matrix during synthesis. In other words, we have shown that the use of an acidic environment in the first stage of synthesis increases the sorption capacity of BSA.

Figure S10 represents a dependence of recovery on the protein:template molar ratio during the synthesis. In our previous paper [15], we suggested that a calculated number of binding sites can be used to select the initial minimal template:protein molar ratio during synthesis. In the present study, we improved the computational model and supported it with experimental methods. The presented model shows the necessity of using a five-fold molar excess of template molecules relative to the calculated number of binding sites for successful synthesis.

## 4. Discussion

Our research investigated the relationship between the fundamental and applied aspects of IP development. By the molecular modeling of several stages of the imprinting process, we showed the structural features of IP formation. The analysis results reveal that the nature of the protein matrix and template molecules are the most important factors for producing efficient IPs. This makes sense because the nature of reactants is directly associated with the sorption capacity of IPs. The different influences of structural factors on the sorption properties of IPs is mainly explained by the ability of the protein matrix to change their conformational state during the imprinting process, thereby providing alternative patterns of ligand–protein interactions and additional binding sites. In this work, we demonstrated that protonation of BSA at the first stage of synthesis leads to an increase in the number of binding sites for 4-HC during the imprinting process. Since each protein has an optimal pH range or temperature within which its binding properties are maximized, the application of computational methodologies in the development of IPs can help screen and select the optimal components for synthesis and explain the optimal molar ratio. Another important advantage of in silico experiments is the ability to theoretically test toxic, unstable, or expensive template molecules before an experiment begins. In this regard, rational design reduces the labor costs of building an IP library and offers a promising direction for increasing practical interest in this receptor production method.

Obviously, multiple factors influence the binding process, including the structural and thermodynamic properties of the solvent, the flexibility of the ligand-binding pocket and the surrounding regions, the molecular structure of the ligand and protein, and the changes in intermolecular forces during IP formation [13]. Free-energy analysis can indirectly highlight structural features of the protein matrix caused by important conformational changes. This may be taken into further consideration when making a final decision regarding the protein matrix state before adding a template. Characterizing the folding/unfolding pathway is a relatively challenging task, especially for multidomain proteins, because each domain is capable of unfolding/refolding separately, and the interaction of different domains within the protein through a variety of short- and long-range interactions can influence the overall folding topology of the protein [61]. In this regard, a comprehensive and detailed theoretical study is essential for a better understanding of the structural features of multidomain proteins and BSA in particular.

## 5. Conclusions

In this work, we used computational methods in combination with experimental techniques to gain a further understanding of the performance of IP formation based on predictions of ligand–protein interactions at the atomic level. A mechanistic study of BSA-4-HC interactions during imprinting as a model system was conducted. The proposed computational method-based methodology effectively helped describe the intermolecular interactions between proteins and template molecules during the imprinting process and can be useful for determining the optimal protein:template molar ratio for producing the most efficient IPs. The theoretical results were successfully verified with experimental data. ITC allowed us to directly measure heat exchange during the association process, determine the forces that drive the binding process, and stabilize intermolecular interactions. Thus, this study represents a step toward the future rational in silico design of IPs for proteins.

## Figures and Tables

**Table 1:** Electrokinetic properties and size of BSA at different pH values.

System	ζ-Potential, mV	$R_{h}$, nm	Polydispersity Index
BSA at pH 8	−19.7	4.05	0.38
BSA at pH 7.4	−10.5	4.70	0.22
BSA at pH 4.5	−4.6	4.75	0.28
BSA at pH 3	15.3	5.75	0.43
BSA at pH 8 + glutaraldehyde	−18.8	9.27	0.55
BSA at pH 8 + 4–HC + glutaraldehyde	−15.5	7.52	0.45

**Table 2:** Diffusion coefficients of BSA at different pH values obtained using DOSY NMR measurements.

pH Value	$D\times 10^{-11}$, m^2^ s^−1^
7.7	5.62
4.7	6.31
3.3	3.98

**Table 3:** The fitting parameters of binding isotherms for 0.5 mg mL^−1^ of 4–HC titration into the 5 mg mL^−1^ of BSA via the multiple-site model at different pH levels at 298 K.

Process	1			2		
pH	3	7.4	9	3	7.4	9
ΔH, kJ mol^−1^	61±40	−11±2	−36±3	175±3	14±1	−84±2
*n*	1.6±0.5	4.7±0.3	4.9±0.2	10.0±0.3	9.1±0.3	9.6±0.8
*K*, $10^{7}$ mol^−1^	99±25	74±40	98±30	6±3	3±2	2±1
ΔS, J mol^−1^ K^−1^	349	134	57	745	188	−147
ΔG, kJ mol^−1^	−43±4	−50±2	−50±3	−47±3	−42±1	−40±2

## Data Availability

All data generated or analyzed during this study are included in the published article and its Supplementary Materials.

## Supplementary Materials

The following supporting information can be downloaded at https://www.mdpi.com/article/10.3390/polym18111280/s1. Figure S1. Distribution of BSA by size at pH 3 (a), 4.5 (b), 7.4 (c), and 8 (d); BSA + Glutaraldehyde (e); BSA + Glutaraldehyde + 4–HC (f) via DLS. Figure S2. DOSY NMR spectra of free BSA at different pH values. Figure S3. DOSY NMR spectra from bottom to top: BSA at pH3, complex BSA—4–HC (Mixture **1B**), complex BSA—4–HC at pH 8–9 (Mixture **2B**), and BSA—4–HC at pH 8–9 after addition of Glutaraldehyde (Mixture **3B**). Figure S4. ATR–FTIR spectra of BSA + glutaraldehyde and BSA + 4–HC + glutaraldehyde mixtures and reference substances. Figure S5. The titration plots, obtained after subtracting the thermograms of 4–HC dilution with buffer solutions at required pH values (3; 7.4 or 9). Figure S6. Absorption spectroscopy of coumarin and 4–HC (a); chromatogram of coumarin and 4–HC at different pH values. Figure S7. BSA-4-HC associate at pH 3. (a) Structure obtained by docking results; (b) stable structure after MD simulation; (c) average RMSD of ligands during MD simulation at pH 3. Ligands 5 and 6 are not presented due to high values of RMSD (more than 40 nm). Figure S8. Transition of BSA—4–HC associate from pH 3 to pH 9. (a) Stable structure after MD simulation at pH 3; (b) stable structure after MD simulation at pH 9; (c) average RMSD of ligands during MD simulation. Figure S9. BSA—4–HC associate at pH 9. (a) Stable structure after MD simulation; (b) average RMSD of ligands during MD simulation; (c) average RMSD of C-alphas of BSA during MD simulation. Figure S10. Dependence of recovery on the protein:template molar ratio during the synthesis of IPs. Table S1. Diffusion coefficients obtained using DOSY NMR measurements in m^2^ × s^−1^. For setails for sample preparation, see the Experimental Section. Table S2. Functional amino acids for BSA-4-HC interactions during the synthesis process before fixing the protein matrix.

## Abbreviations

The following abbreviations are used in this manuscript:

4–HC	4-hydroxycoumarin;
BSA	Bovine serum albumin;
DLS	Dynamic light scattering;
DOSY	Diffusion-ordered NMR spectroscopy;
IPs	Imprinted proteins;
ITC	Isothermal titration calorimetry;
MD	Molecular dynamics;
MM	Molecular mechanics;
MM–GBSA	Molecular-mechanics generalized-Born surface area;
QM	Quantum mechanics.
