# Supplementary material for: Toward Rational Design of Imprinted Proteins Based on Albumins: Computational and Experimental Studies
Source: Polymers (Basel). 2026 May 23;18(11):1280. doi: 10.3390/polym18111280 (PMC13259328; doi:10.3390/polym18111280)
Supplement: Supplementary file 1 [file polymers-18-01280-s001.zip › polymers-4270799-supplementary.pdf]

Toward Rational Design of Imprinted Proteins based on Albumins: Computational and Experimental Studies

Polina M. Ilicheva[a]*, Alexander L. Kwiatkowski[b], Ivan A. Reshetnik[a], Kirill Y. Presnyakov[a], Ilya E. Menyailo[a], Mikhail V. Pozharov[a], Pavel S. Pidenko[a], Yulia B. Monakhova[a,c], Olga E. Philippova[b], Natalia A. Burmistrova[a]

[a] *Institute of Chemistry, Saratov State University, 410012, Saratov, Russia*
[b] *Physics Department, Lomonosov Moscow State University, 119991, Moscow, Russia*
[c] *Department of Chemistry and Biotechnology, University of Applied Sciences Aachen, D-52428, Jülich, Germany*

*Corresponding author(s). E-mail(s): naburmistrova@mail.ru, ilichevapm@gmail.com;

MATERIALS AND METHODS

The purity of all chemicals was analytical grade.

Preparation of Pauly's reagent

The Pauly's reagent was synthesized by mixing two components — (i) a solution of 20 mg of 4-aminobenzenesulfonic acid in 5 ml of 0.32 M solution of HCl, and (ii) a solution of 200 mg $NaNO_2$ in 40 mL of Milli-Q water — at a ratio of 200:1 (v/v). The mixture was incubated for 30 minutes in a refrigerator at 4°C.

Determination of o-coumaric acid

The presence of o-coumaric acid in coumarin and 4-hydroxycoumarin (4–HC) solution was determined through colorimetry and HPLC–UV. In the case of colorimetric detection, 40 μL of freshly prepared Pauly's reagent were added to coumarin and 4-HC solutions (100 μg/mL, pH 5 and 9) and incubated for 5 minutes; after that, absorption spectra were registered for 200–600 nm wavelength range via Shimadzu UV–1800 Spectrometer (Shimadzu Co, Kyoto, Japan). HPLC–UV was performed via LC–20AD high-performance liquid chromatography with UV detector (Shimadzu Co, Kyoto, Japan) in isocratic mode at a flow rate of 1.0 mL at a constant temperature of 25 °C with mobile phase A (Milli–Q water) and mobile phase B (acetonitrile) taken at a ratio of 40 : 60; retention times for coumarine and 4–HC were 2 min 15 s and 2 min 10 s, respectively. Absorbance signal was recorded at 251 nm.

Infrared spectroscopy

To further confirm that ligands and glutaraldehyde were interacted with BSA, we studied the samples (mix BSA + glutaraldehyde; mix BSA + 4–HC + glutaraldehyde) and reference substances (BSA, 4–HC, glutaraldehyde) with ATR–FTIR spectroscopy (FT–801 infrared Fourier spectrometers with attenuated total reflection attachments, Simex, Novosibirsk Russia). The sample (30 μL) was dried with an air stream on the diamond crystal surface, and the spectrum was recorded. All spectra were subjected to CO_2 peak subtraction, baseline correction, and normalization.

RESULTS

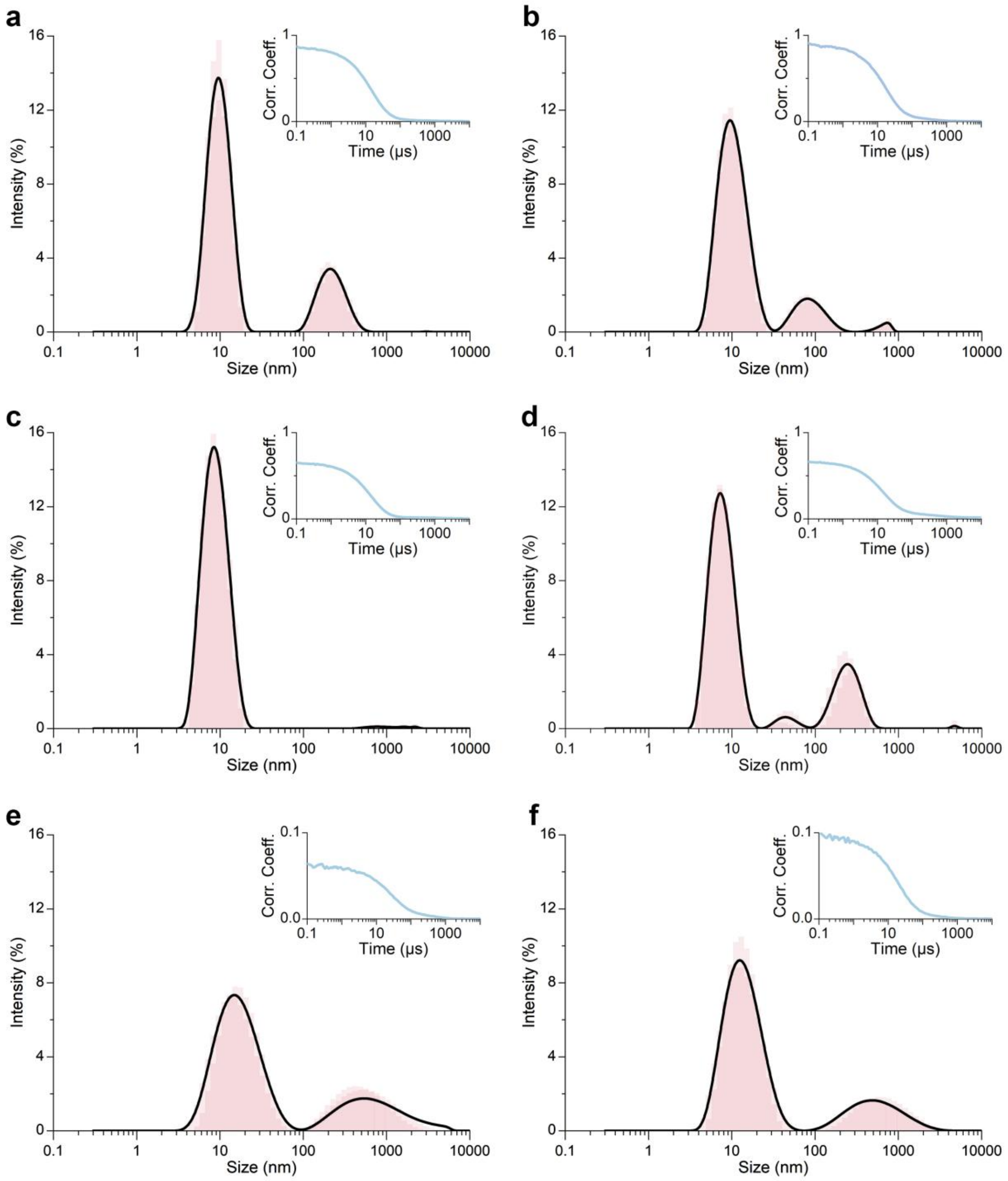

Figure S1. Distribution of BSA by the size at pH 3 (a), 4.5 (b), 7.4 (c), 8(d); BSA + Glutaraldehyde (e); BSA + Glutaraldehyde + 4–HC (f) via DLS

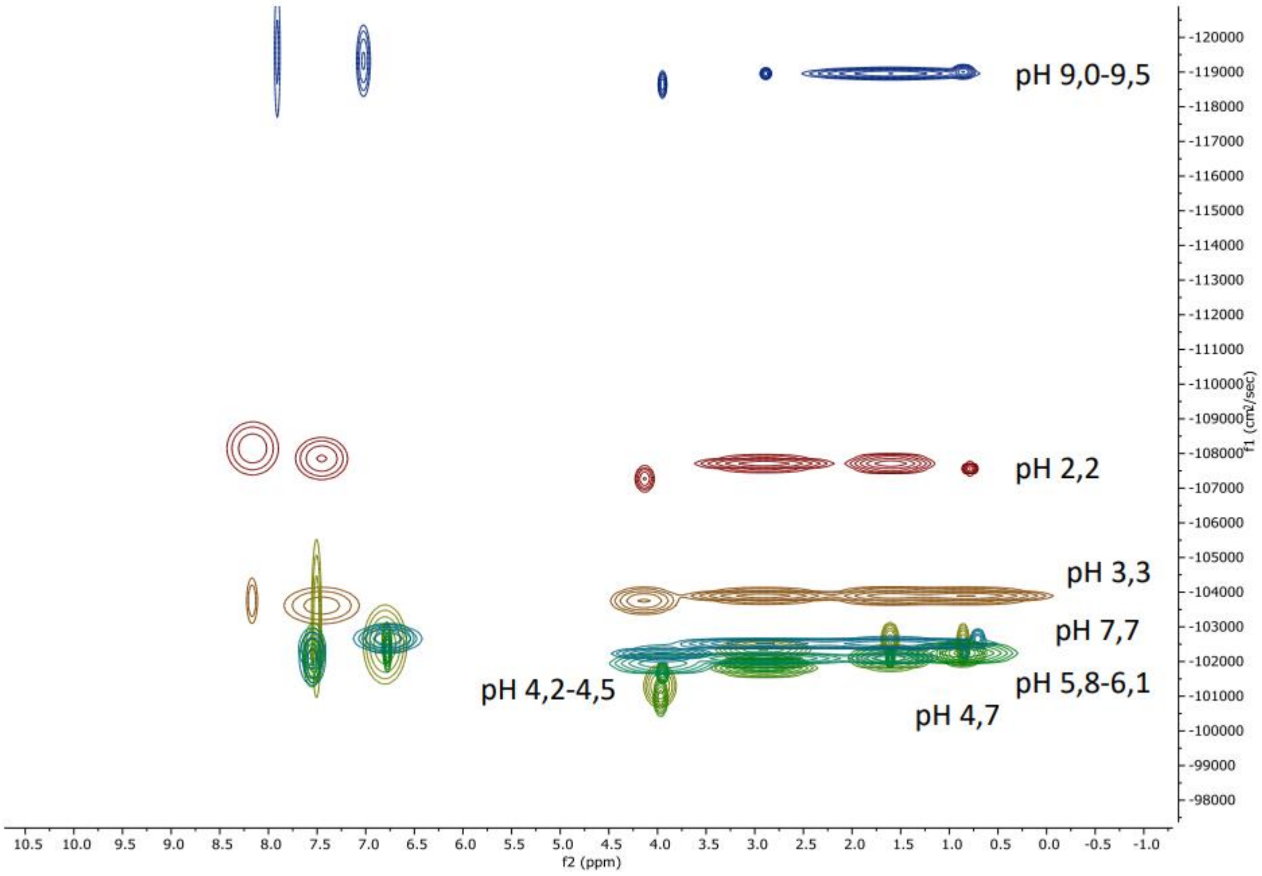

Figure S2. DOSY NMR spectra of free BSA at different pH values.

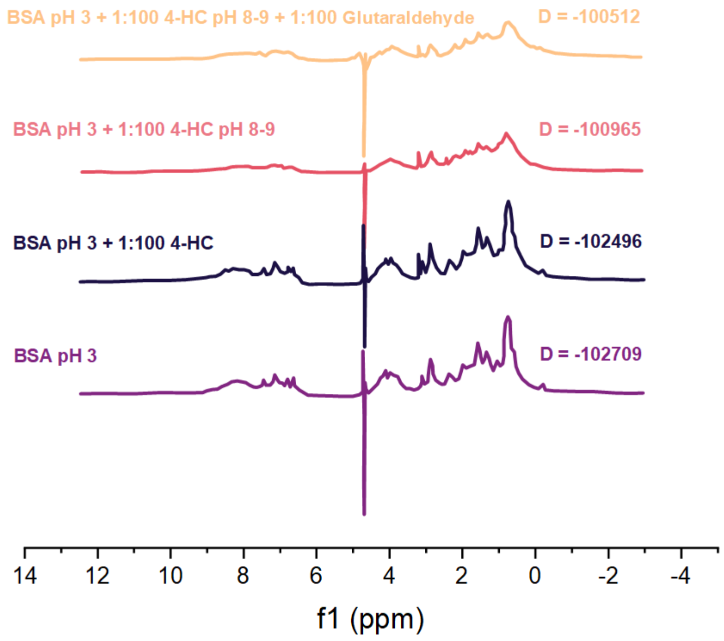

Figure S3. DOSY NMR spectra from bottom to top: BSA at pH3, complex BSA—4–HC (Mixture **1B**), complex BSA—4–HC at pH 8–9 (Mixture **2B**) and BSA—4–HC at pH 8–9 after addition of Glutaraldehyde (Mixture **3B**).

Table S1. Diffusion coefficients obtained using DOSY NMR measurements in $m^2 \times s^{-1}$. Details for sample preparation see in experimental section.

	**BSA**<br><br>**pH 3**	**BSA + 4–HC**<br>**(Mixtures 1A–1C)**	**BSA + 4–HC**<br>**(Mixtures 2A–2C)**<br>**pH 8–9**	**BSA + 4–HC + GA**<br>**(Mixtures 3A–3C)**<br>**pH 8–9**
BSA	5.37	–	–	–
A	–	5.62	7.41	9.12
B	–	5.62	7.94	8.91
C	–	5.62	8.13	7.76

After the synthesis of IPs in the presence of 4–HC, the appearance and transformation of bands (Figure S10) at 755 (C—H out of plane), 774 (C—H out of plane), 1192 (C—O stretch), 1238 (C—O stretch), 1274, 1532 (NH out of plane), 1574 (transformation) cm^{-1} are observed. The addition of glutaraldehyde leads to the appearance of bands at 1047, 1715 (C=O stretch) cm^{-1} relative to native BSA.

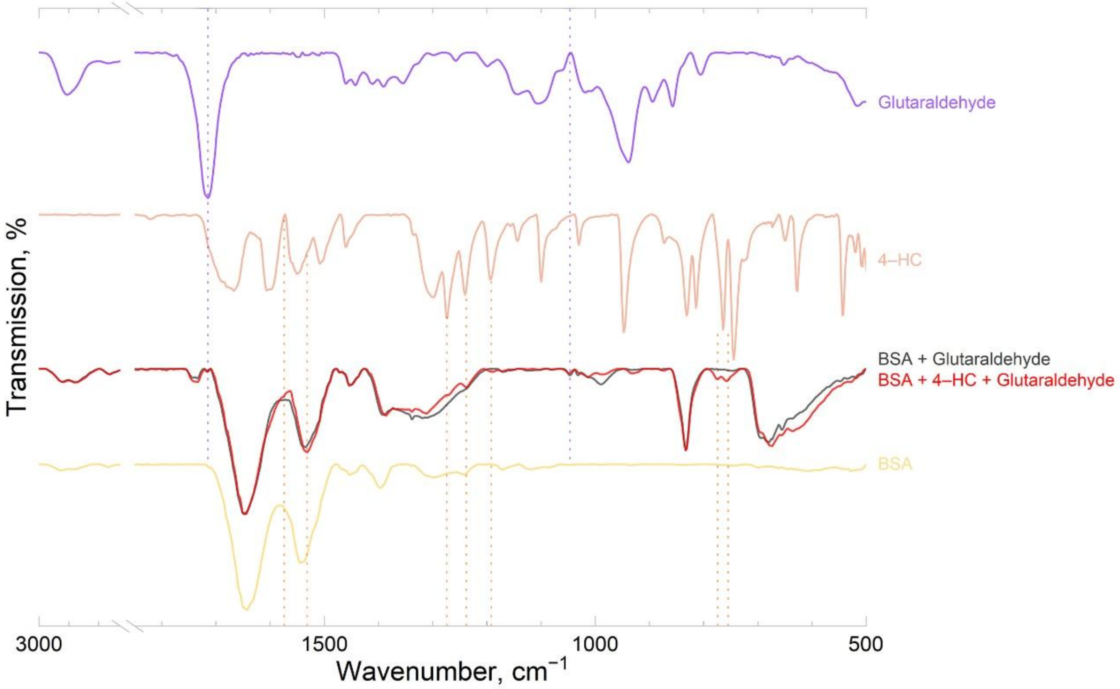

Figure S4. ATR–FTIR spectra of mixes BSA + glutaraldehyde and BSA + 4–HC + glutaraldehyde and reference substances

Table S2. Functional amino acids for BSA-4-HC interactions during the synthesis process before fixing the protein matrix.

Ligand	Amino acids	$\Delta G_{MM/GBSA}$, kcal/mol	Binding site
Site 1	L42, Pi-Pi (F27)	−39	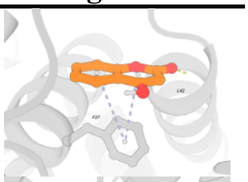
Site 2	Q29, D107, Pi-Pi (Y30, Y147)	−40	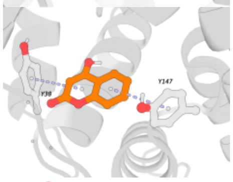
Site 3	E351, T355, Pi-pi (F329)	−38	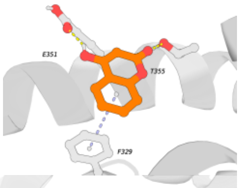
Site 4	Y149, R256	−31	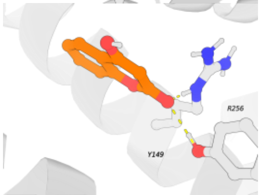
Site 5	E441, Pi-cat (K388, R444)	−21	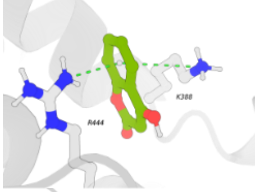
Site 6	P572	−20	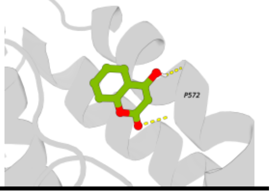

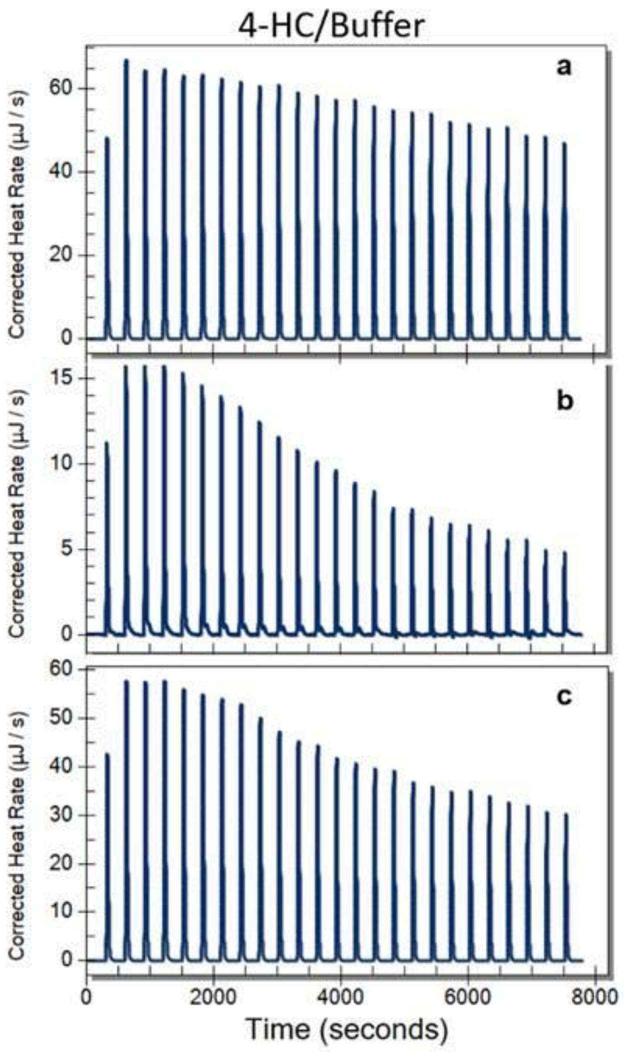

Figure S5. The titration plots, obtained after subtracting the thermograms of 4–HC dilution with buffer solutions at required pH values (3; 7.4 or 9).

After Pauly's reagent was added to alkaline coumarin solution, the solution's color turned yellow, and a characteristic band (peaking at 456 nm) appeared in the absorption spectrum. Such changes were not observed for 4–HC solution (Fig. S9a). Additional HPLC–UV assay was performed to confirm the formation of hydrolysis products (Fig. S9b,c). We found that in alkaline coumarin solution (pH 9) there were two chromatographic peaks with retention times of 1 min 6 s and 2 min 15 s, respectively. As peak at 2 min 15 s is characteristic for coumarin; the additional peak corresponds to o-coumaric acid. As for both 4–HC solutions (pH 5 and 9), there was only one chromatographic peak at 2 min 10 s. The results of NMR-spectroscopic assay of solution pH influence on the structure of 4–HC also showed that there were no changes in 4–HC molecule structure. Thus, we can conclude that 4–HC does not undergo hydrolysis in IPs synthesis conditions.

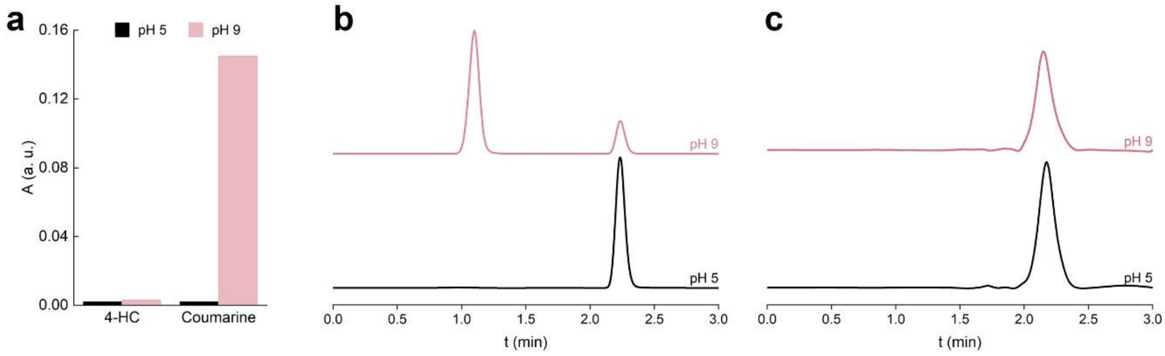

Figure S6. Absorption spectroscopy of coumarin and 4–HC (a); Chromatogram of coumarin and 4–HC at different pH values.

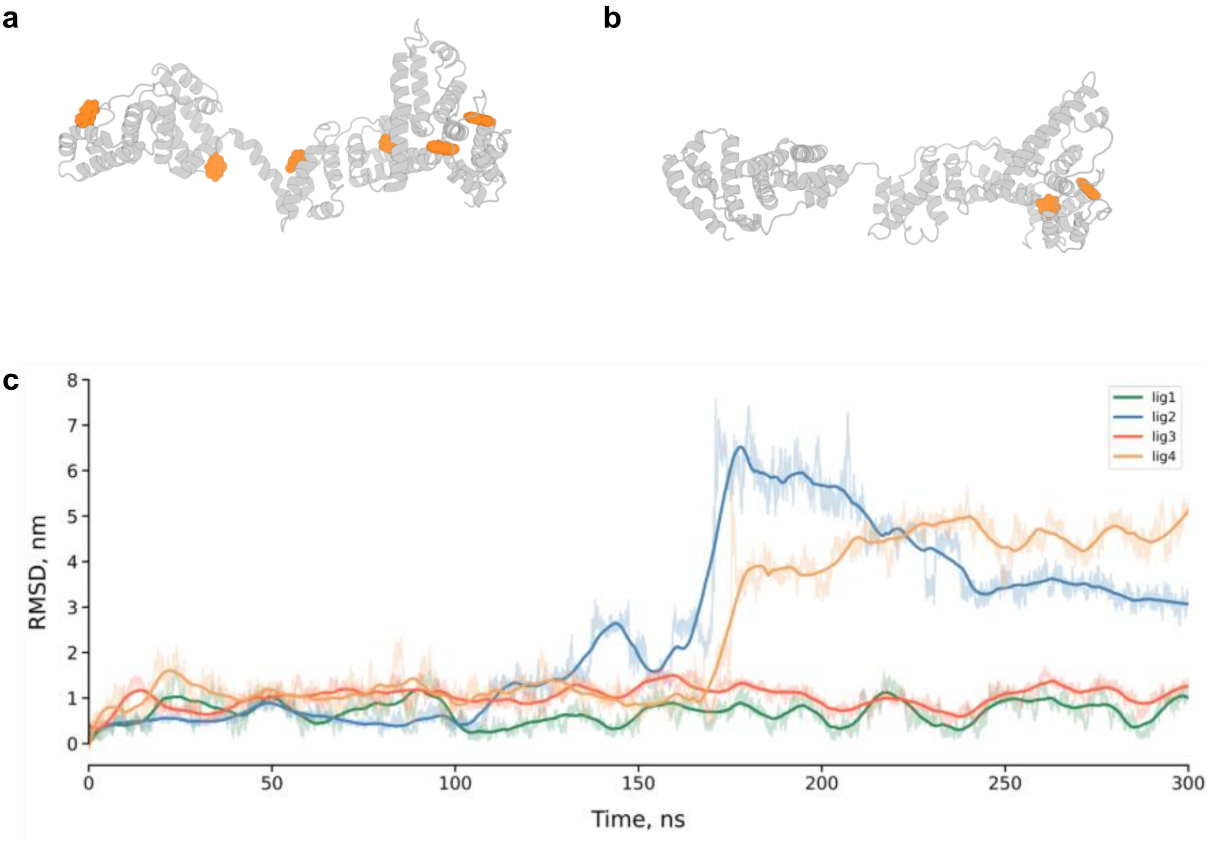

Figure S7. BSA-4-HC associate at pH 3. (a) — Structure obtained by docking results; (b) — Stable structure after MD simulation; (c) — Average RMSD of ligands during MD simulation at pH 3. Ligands 5 and 6 are not presented due to high values of RMSD (more than 40 nm).

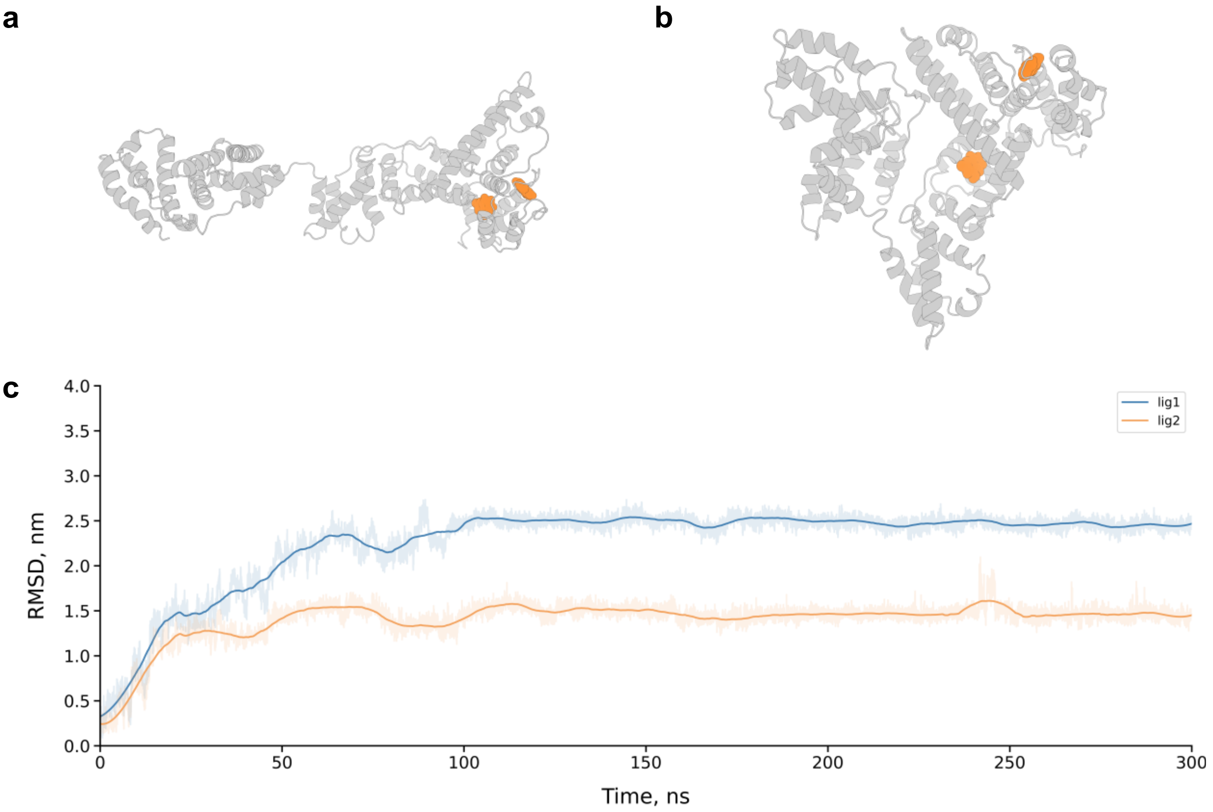

Figure S8. Transition of BSA—4–HC associate from pH 3 to pH 9. (a) — Stable structure after MD simulation at pH 3; (b) — Stable structure after MD simulation at pH 9; (c) — Average RMSD of ligands during MD simulation.

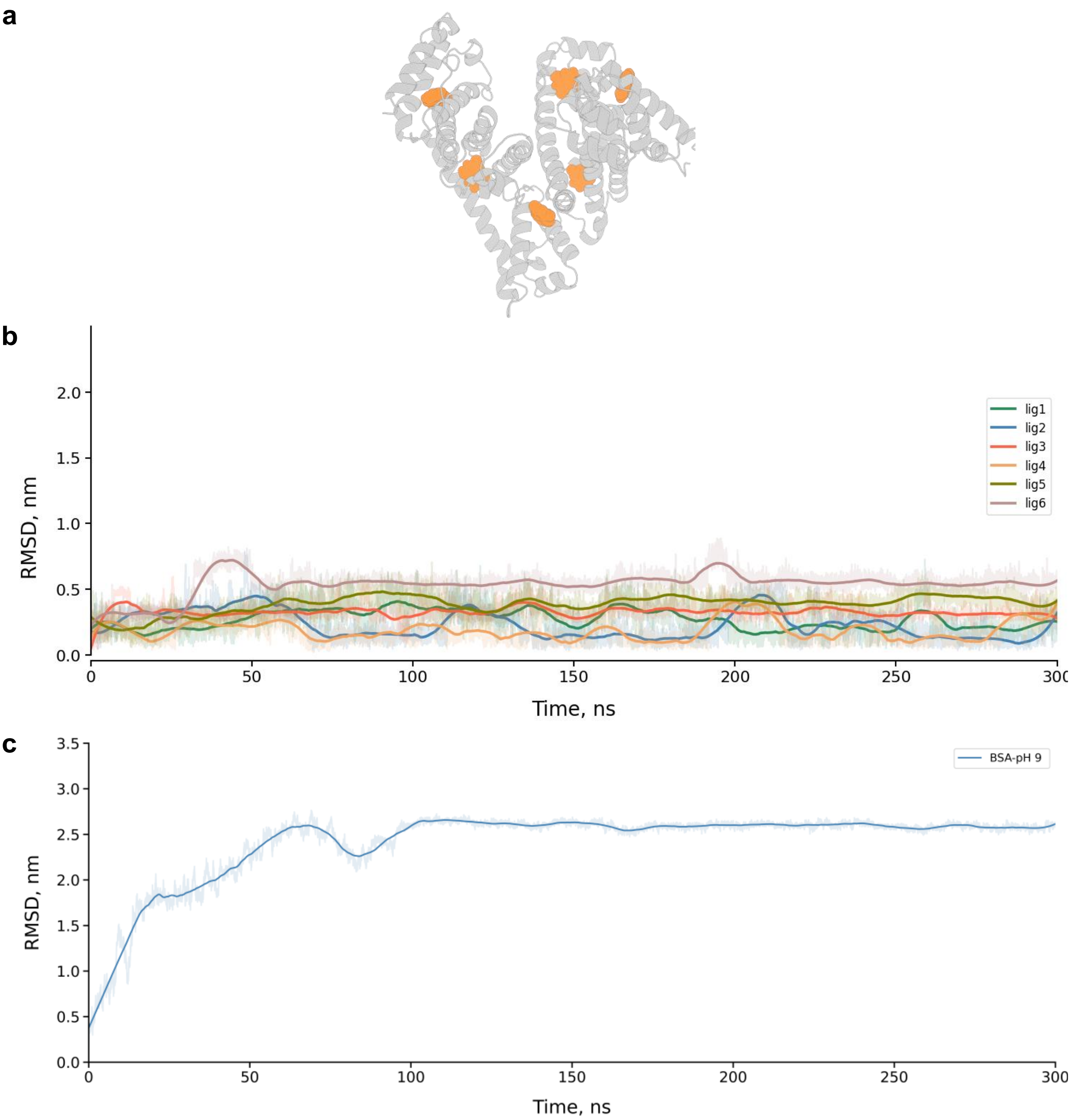

Figure S9. BSA—4–HC associate at pH 9. (a) — Stable structure after MD simulation; (b) — Average RMSD of ligands during MD simulation. (c) — Average RMSD of C-alphas of BSA during MD simulation.

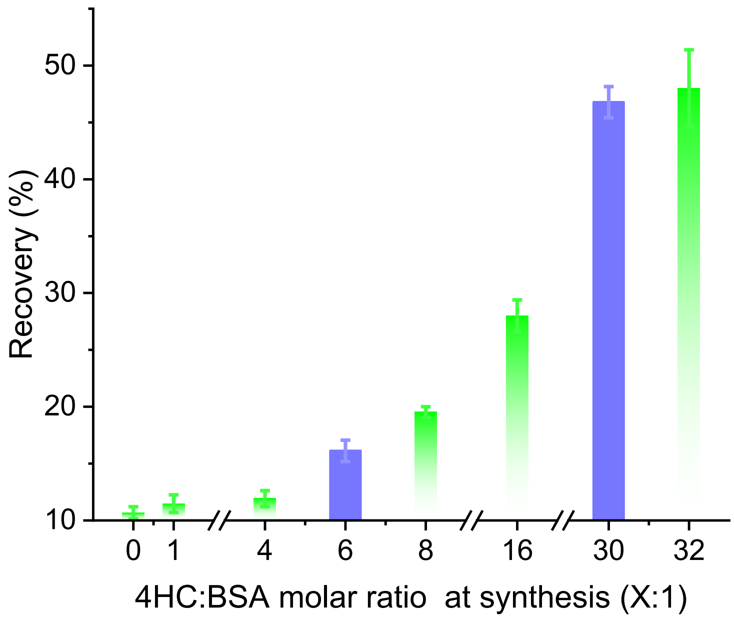

Figure S10. Dependence of recovery on the protein:template molar ratio during the synthesis of IPs
